# Singapore multidisciplinary consensus recommendations on muscle health in older adults: assessment and multimodal targeted intervention across the continuum of care

**DOI:** 10.1186/s12877-021-02240-8

**Published:** 2021-05-17

**Authors:** Samuel T. H. Chew, Geetha Kayambu, Charles Chin Han Lew, Tze Pin Ng, Fangyi Ong, Jonathan Tan, Ngiap Chuan Tan, Shuen-Loong Tham

**Affiliations:** 1 Department of Geriatric Medicine, Changi General Hospital, 2 Simei Street 3, Singapore, 529889 Singapore; 2 Society for Geriatric Medicine Singapore, Singapore, Singapore; 3 Department of Rehabilitation, National University Hospital, Singapore, Singapore; 4 Singapore Nutrition and Dietetics Association, Singapore, Singapore; 5 Yong Loo Lin School of Medicine, National University of Singapore, Singapore, Singapore; 6 Mount Elizabeth Novena Hospital, Singapore, Singapore; 7 Department of Research, SingHealth Polyclinics, Singapore, Singapore; 8 Department of Rehabilitation Medicine, Tan Tock Seng Hospital, Singapore, Singapore; 9 Society of Rehabilitation Medicine, Singapore, Singapore

**Keywords:** Muscle, Sarcopenia, Older adult, Multidisciplinary, Exercise, Nutrition, Consensus

## Abstract

**Background:**

The rapidly aging societies worldwide and in Singapore present a unique challenge, requiring an integrated multidisciplinary approach to address high-value targets such as muscle health. We propose pragmatic evidence-based multidisciplinary consensus recommendations for the assessment and multi-modal management of muscle health in older adults (≥65 years) across the continuum of care.

**Methods:**

The recommendations are derived from an in-depth review of published literature by a multidisciplinary working group with clinical experience in the care of the older population in both acute and community settings.

**Results:**

The panel recommends screening for muscle impairment using the SARC-F questionnaire, followed by assessment for low muscle strength (handgrip strength or 5-times chair stand test ≥10 s as a surrogate for lower limb strength) to diagnose possible/probable sarcopenia. For uncomplicated cases, lifestyle modifications in exercise and diet can be initiated in the community setting without further assessment. Where indicated, individuals diagnosed with possible/probable sarcopenia should undergo further assessment.

Diagnosis of sarcopenia should be based on low muscle strength and low muscle mass (bioimpedance analysis, dual-energy X-ray absorptiometry or calf circumference as a surrogate). The severity of sarcopenia should be determined by assessment of physical performance (gait speed or 5-times chair stand test ≥12 s as a surrogate for gait speed). To treat sarcopenia, we recommend a combination of progressive resistance-based exercise training and optimization of nutritional intake (energy, protein and functional ingredients). High quality protein in sufficient quantity, to overcome anabolic resistance in older adults, and distributed throughout the day to enable maximum muscle protein synthesis, is essential. The addition of resistance-based exercise training is synergistic in improving the sensitivity of muscle protein synthesis response to the provision of amino acids and reducing anabolic resistance. An expected dose-response relationship between the intensity of resistance-based training, lean mass and muscle strength is described.

**Conclusions:**

Reviewed and endorsed by the Society of Rehabilitation Medicine Singapore and the Singapore Nutrition and Dietetics Association, these multidisciplinary consensus recommendations can provide guidance in the formulation of comprehensive and pragmatic management plans to improve muscle health in older adults in Singapore and Asia.

## Background

Aging is accompanied by a loss of muscle health which includes progressive loss of muscle mass and function [1–3]. Loss of muscle mass and function are associated with negative outcomes such as clinical complications, lower physical function, poor quality-of-life, longer length of hospitalization, morbidity and mortality, and higher healthcare costs [4–7]. For the purpose of this paper, muscle health is defined as having adequate muscle mass and muscle function.

Decline in function (defined as muscle strength and/or physical performance) and muscle mass are the defining features of sarcopenia [8–10]. These are predominantly associated with aging (primary sarcopenia), while systemic disease, physical inactivity or malnutrition are implicated in secondary sarcopenia [9, 10]. Sarcopenia is estimated to affect approximately 10% of community-dwelling older adults globally [11] and is prevalent in up to 33% of frail older adults in nursing homes [12] and 22–26% of older adults in inpatient settings [13].

In Singapore, 44% of adults ≥65 years old attending specialist outpatient clinics were at risk of sarcopenia based on the SARC-F questionnaire [14]. Sarcopenia, diagnosed using the Asian Working Group for Sarcopenia (AWGS) 2014 criteria, was reported in 25% of community-dwelling and functionally independent older adults aged ≥50 years [15] and 27.4% of older adults (60–78 years old) with type 2 diabetes in a primary care setting [16]. In a cross-sectional study of 811 community-dwelling independently ambulant older adults > 65 years old at risk of malnutrition (defined by Malnutrition Universal Screening Tool score ≥ 1) in Singapore, the prevalence of sarcopenia based on the AWGS 2014 criteria increased significantly to 70% [17]. In addition, these subjects with sarcopenia were found to have lower total energy intake and energy-adjusted protein intake based on 24-h dietary recall [17]. This study also reported a prevalence of 81.3% for low appendicular skeletal mass index (ASMI) based on the AWGS 2014 cut-offs [17], a striking four-fold increase compared with a nourished cohort with similar baseline characteristics where the prevalence for low ASMI was only 20.6% [18]. These findings are concerning as one in two Singaporeans aged > 65 years is expected to become severely disabled and require long-term care in their lifetime [19].

The importance of muscle health remains under-recognized across healthcare settings [20–22]. An international survey found that only 50% of clinicians measured at least one muscle parameter in patients aged ≥60 years [23]. Majority of healthcare professionals (HCPs) lack guidance on the recognition, assessment and management of muscle health [20, 24]. Other barriers include time constraints; complexity of measurement variables; a paucity of population-specific cut-offs for these measures; and lack of collaboration [9, 22, 24].

Several international and regional guidelines and consensus recommendations on sarcopenia and muscle health are available [9, 10, 25], including the recently revised AWGS 2019 Consensus Update on Sarcopenia Diagnosis and Treatment (AWGS 2019), which also provided cut-off values for the screening and diagnosis of sarcopenia based on Asian patients [26]. While some of these guidelines provide recommendations to prevent and improve sarcopenia [10, 25, 26], they may be too broad or too specific in nature for use in the clinical setting directly. We aim to bridge this gap through a multidisciplinary review using a Delphi-like approach to meet the needs for concise and pragmatic consensus recommendations for optimizing muscle health in older adults aged ≥65 years in Singapore. We aim to complement the recommendations from AWGS 2019 by proposing an additional surrogate measure for lower limb strength, as well as providing a more detailed and targeted approach in terms of interventions to improve nutrition and muscle health in older patients. We also aim to provide more input from a multidisciplinary and holistic point of view, and to propose some population-based strategies which will complement the strength of the multiple stakeholders involved in the care of this sub-group of older people in Singapore who may be at risk of poor muscle health. These recommendations are intended for HCPs in the clinical settings across the continuum of care in the community, outpatient, inpatient and long-term care settings.

## Methods

These recommendations were developed by a multidisciplinary working group of eight senior clinicians and researchers in geriatrics, dietetics, gerontology, intensive care medicine, family medicine, physiotherapy and rehabilitation medicine to encompass the entire continuum of care.

STH Chew is a Senior Consultant Geriatrician and the Principal Investigator for the SHIELD study [17, 18] in community dwelling older adults in Singapore, G Kayambu (PhD) is a Senior Physiotherapist and Director for Departmental Research, National University Hospital Singapore, C Lew (PhD) is a Principal Dietitian and Researcher in Nutrition, TP Ng is a Medical Epidemiologist in Academia, Researcher and the Principle Investigator for the Singapore Longitudinal Ageing Study (SLAS, ClinicalTrials.gov Identifier: NCT03405675), F Ong is a Principal Dietitian with extensive clinical experience supporting nutrition in older patients across the continuum of care, J Tan is a Senior Consultant Anaesthetist, a founding member of and the current Scientific Chair for the Singapore Society for Parenteral and Enteral Nutrition (SingSPEN), NC Tan is a Senior Consultant Family Physician, clinician-innovator cum the Director of Research for Singhealth Polyclinics with funded studies on sarcopenia in the geriatric population, and SL Tham is a Consultant and Program Director for Rehabilitation Medicine at Tan Tock Seng Hospital.

STH Chew, G Kayambu, C Lew and F Ong, and SL Tham were nominated to represent the Society of Geriatric Medicine Singapore, Singapore of Physiotherapy Association, Singapore Nutrition and Dietetics Association, and Singapore Rehabilitation Association respectively.

The panel convened at a face-to-face meeting in Singapore in late-April 2019 to review and discuss available evidence, share clinical practice experience on the management of muscle health and forge a way forward on how best to apply the current evidence and guidelines in clinical settings. We defined the clinical problem as the following: *“In light of the rapidly ageing population in Singapore and Asia, what are the best evidence-based interventions available to optimise muscle health in older adults >65 years old that can be delivered by a multidisciplinary team across the continuum of care?”*

Prior to the meeting, a targeted literature search using PubMed was conducted with the following keywords and their combinations: sarcopenia, muscle health, elderly, nutrition, protein, vitamin, oral nutritional supplements (ONS), β-hydroxy β-methylbutyrate (HMB), exercise, guidelines, consensus, Asia and Singapore. Draft recommendations were subsequently developed based on major international guidelines on sarcopenia and nutrition [8, 9, 12, 20, 25, 27], as well as relevant evidence identified from the literature. Our concise and harmonized operational definition and diagnostic criteria for sarcopenia, using SARC-F, muscle strength, muscle mass and physical performance in a step-wise manner, are derived from definitions and criteria proposed by the recently published second European Working Group on Sarcopenia in Older People (EWGSOP2) and AWGS 2019 [9, 26]. The treatment recommendations by AWGS 2019 are themselves guided by the systematic review by Arai et al. [10]. Where available, we have adopted the cut-off values recommended by the AWGS 2019 [26]. Otherwise, cut-off values from international literature are used.

Following an in-depth discussion at the initial meeting, multiple rounds of e-mail correspondence until 100% agreement was achieved for each section in an iterative manner took place over the next 6 months. We have also included the updated recommendations from the AWGS 2019 as well as other recent relevant publications. The finalized consensus recommendations with approval from all authors on screening and diagnosis of sarcopenia; physical activity; nutritional interventions (protein, vitamin D, HMB, ONS); and education (patient and HCP) are presented in this paper.

These recommendations have been reviewed and endorsed by the Society of Rehabilitation Medicine Singapore and the Singapore Nutrition and Dietetics Association. The Singapore Physiotherapy Association has also reviewed and provided invaluable input on the recommendations, particularly in the sections on interventions for muscle health, the structured resistance exercise training (RET)-based exercise program, and population-based strategies for muscle health.

## Results

Tables 1 and 2 lists the recommendations developed based on international and regional evidence, and clinical perspectives contextualized to Singapore.

**Table 1:** Summary of sconsensus recommendations on screening and diagnosis for muscle health

Management of muscle health across the continuum of care in Singapore – screening and diagnosis for muscle health	
1. The importance of muscle health (mass, strength, and function) should be emphasized across the continuum of care.	
2. Muscle health should be prioritized in older adults (≥65 years), particularly in individuals with conditions who may be at risk of sarcopenia.	
3. In most settings, screening for possible/probable sarcopenia or muscle impairment can be performed using the SARC-F questionnaire, followed by the assessment for low muscle strength via handgrip strength or the 5-times chair stand test with a cut-off of ≥10 s as surrogate measures of muscle strength.	
4. Diagnosis of confirmed sarcopenia should be based on the presence of low muscle strength *and* low muscle mass; for severe sarcopenia, low physical performance is also present in addition to low muscle strength *and* low muscle mass.	
5. In community and outpatient settings, bioimpedance analysis can be used to determine low muscle mass. In the absence of other alternatives, calf circumference can be used as a surrogate measure of muscle mass in patients without edema and not suspected to have sarcopenic obesity. In inpatient settings, dual-energy X-ray absorptiometry can be used as the reference standard for measuring muscle mass.	
6. Physical performance can be measured using the usual gait speed or by using the 5-times chair stand test with a cut-off of ≥12 s as a surrogate measure of gait speed of 1.0 m/s.

**Table 2:** Summary of consensus recommendations on interventions for maintaining muscle health

Management of muscle health across the continuum of care in Singapore – recommended interventions for muscle health	
7. Progressive resistance/weight-based training is effective for improving muscle mass, strength and physical performance	
8. Adequate calorie and protein diet support muscle health for healthy community-dwelling older adults.	
9. Supplementation of protein and calories, either via whole foods and/or high protein oral nutrition supplements, should be the primary focus of any nutrition interventions aimed at optimizing muscle health and recovery in hospitalized patients.	
10. Meeting the recommended daily intake of vitamin D (600–800 IU) may improve muscle strength across the continuum of care and vitamin D deficiency should be treated.	
11. A combination of physical activity and nutritional interventions is strongly recommended for optimal muscle health in patients with malnutrition and at risk of malnutrition.	
12. A multidisciplinary team (physicians, dietitians/nutritionists, physiotherapists, nurses and other relevant healthcare professionals) is essential for optimizing muscle health in all settings.	
13. Patients and caregivers should be educated on the importance of combining physical activity and nutritional interventions for improving muscle health.	
14. Across all healthcare settings, urgent initiatives are required to raise awareness, educate and skill relevant healthcare professionals on screening, prevention and management of poor muscle health.	

### Importance of muscle health

#### Recommendation 1

**The importance of muscle health (mass, strength, and function) should be emphasized across the continuum of care.**

Impaired muscle health contributes to adverse clinical and functional outcomes in inpatient, outpatient and long-term care settings [6, 28]. Impairments in muscle mass and function increase the risk of comorbidities [14, 29, 30]; functional decline and physical disability [31, 32]; risk of falls [14, 33, 34]; and loss of independence in activities of daily living [35].

Sarcopenia and measures of muscle function have been shown to predict the risk of mortality in older individuals in the community, inpatient settings and nursing homes [36–40]. Sub-optimal muscle health is associated with a greater risk of institutionalization, rate and length of hospitalization and re-admissions, and polypharmacy [14, 40–43], with a consequent increase in healthcare utilization and expenditure [44–46].

### Screening muscle health across the continuum of care

#### Recommendation 2

**Muscle health should be prioritized in older adults (≥65 years), particularly in individuals with conditions who may be at risk of sarcopenia.**

From the age of 40, about 8% of lean body mass (LBM) loss per decade is expected. This increases to about 15% per decade after age 70; by the age of 80, there is an expected loss of up to 39% of LBM from peak LBM [47–49]. We therefore propose prioritizing muscle health before the onset of peak muscle loss. Muscle health should be re-evaluated after major acute illness or prolonged hospitalization, as the increased inflammatory burden and bed rest during these states may precipitate the loss of muscle mass, strength and functional capacity in older adults [13, 50].

We support the findings of the taskforce for the International Clinical Practice Guidelines for Sarcopenia, which recommends annual screening and additional screening after major health events in individuals > 65 years of age [25]. Further case finding and prioritization of screening can be undertaken in patients with conditions associated with sarcopenia such as old age, frailty, malnutrition, chronic illnesses, cognitive impairment and recurrent falls [26].

#### Recommendation 3

**In most settings, screening for possible/probable sarcopenia or muscle impairment can be performed using the SARC-F questionnaire, followed by the assessment for low muscle strength via handgrip strength or the 5-times chair stand test with a cut-off of ≥10 s as surrogate measures of muscle strength.**

Across all healthcare settings, screening for muscle health and sarcopenia should be rapid and convenient. In line with recent guidelines, we suggest using the SARC-F questionnaire for screening, followed by the assessment for low muscle strength via handgrip strength or the 5-times chair stand test (5CST) as a surrogate measure of lower limb strength [9, 51, 52]. Recommended cut-off values are presented in Table 3. The 5-item SARC-F questionnaire has been shown to be useful in Asian older adults [55–58], with a score of ≥4 predictive of adverse functional outcomes [53]. The SARC-F demonstrates high specificity, but has low sensitivity [58, 59]. Handgrip strength correlates well with lower limb strength and is widely used as a measure of overall strength in studies on sarcopenia and frailty [60]. Specific measurement techniques are required for accurate measurements. The recommended cut-off values for Asian population are < 28 kg for males and < 18 kg for females [26].

**Table 3:** Recommended tools for assessment of muscle health with cut-off values for the Singapore population

Parameter	Recommended tool	Recommended cut-off value for low muscle parameters	Reference
Physical activity related to muscle	SARC-F questionnaire	Score of ≥4 out of 10, indicative of sarcopenia	Malmstrom et al., 2016 [53]; AWGS 2019 [26]
Muscle strength	Handgrip strength	**Men**: < 28 kg **Women**: < 18 kg	AWGS 2019 [26]
	5-times CST test (surrogate measure)	≥10s for 5 rises	Makizako et al., 2017 [54]
Muscle mass	BIA (ASMI)	**Men**: < 7.0 kg/m^2^ **Women**: < 5.7 kg/m^2^	AWGS 2019 [26]
	DXA (ASMI)	**Men**: < 7.0 kg/m^2^ **Women**: < 5.4 kg/m^2^	AWGS 2019 [26]
	Calf circumference (surrogate measure)	**Men**: < 34 cm **Women**: < 33 cm	AWGS 2019 [26]
Physical performance	Usual gait speed	< 1.0 m/s	AWGS 2019 [26]
	5-times CST test^a^ (surrogate measure)	≥12 s as a proxy for low gait speed (< 1.0 m/s)	AWGS 2019 [26]

^a^The use of a standardized protocol, such as the one from the American Academy or Orthotists & Prosthetists is recommended. *Abbreviations*: *ASMI* Appendicular skeletal mass index (ASM adjusted for height), *AWGS* Asian Working Group for Sarcopenia, *BIA* Bioimpedance analysis, *DXA* Dual-energy X-ray absorptiometry, *CST* Chair Stand Test

In the AWGS 2019 guidelines, handgrip strength is the sole measure of strength [26]. However, the chair stand test (CST) can also be used as a proxy for lower leg strength [9]. The EWGSOP2 recommends the 5CST test, which measures the *time* to stand up from a standard chair five times, as a surrogate measure of muscle strength in the lower limbs [9]. The 5CST fulfils an important role as a surrogate measure of lower limb strength for older persons, particularly where the older patient has difficulties following accurately the protocol for hand grip strength measurements, or have joint pathology in their hands precluding the use of a dynamometer. A prospective cohort study of 4335 older people age ≥ 65 living in the community in Japan reported a cut-off of ≥10s for 5CST using receiver operating characteristic (ROC) curve analysis to maximize sensitivity and specificity in predicting future disability [54]. In view of the recognized ethnic variations in normative values for 5CST between Asia and North America populations [61], we will be adopting the 5CST cut-off of ≥10s for our consensus statement.

Patients who screen positive by SARC-F and have low muscle strength would fulfill the criteria for possible/probable sarcopenia. Lifestyle changes and education in terms of exercise and nutrition should be initiated at this point in the community and primary care settings [9, 26]. Where indicated, these patients should be referred for further assessment to confirm the diagnosis of sarcopenia and to identify underlying causes which may be reversible.

#### Recommendation 4

**Diagnosis of confirmed sarcopenia should be based on the presence of low muscle strength and low muscle mass; for severe sarcopenia, low physical performance is also present in addition to low muscle strength and low muscle mass.**

As per the EWGSOP2 and AWGS 2019 consensus, a diagnosis of sarcopenia should be based on the detection of low muscle strength and low muscle mass [9, 26]. Muscle strength is prioritized as a primary determinant of sarcopenia as aging is associated with significantly greater declines in muscle strength and physical performance than in muscle mass [1–3, 62]. Muscle strength has also been shown to predict adverse outcomes, such as falls, functional decline, cardiovascular disease and mortality in older adults [63–67] and reported to be the most reliable predictor of muscle function [9]. In a recent literature review initiated by the World Health Organisation (WHO), physical performance is separate from muscle function, and is defined as an objectively measured whole body function related with mobility [60]. As such, we recommend the assessment for low physical performance as a marker of severe sarcopenia, in the presence of both low muscle strength *and* low muscle mass. The three major causes of secondary sarcopenia are physical inactivity, malnutrition and diseases which lead to inflammation, chronic neurological disorder and musculoskeletal disorder [9], of which the first two are modifiable.

Physical inactivity, due to a sedentary lifestyle or prolonged bed rest, is associated with the development of sarcopenia in older adults [68–70]. Additionally, older age is often characterized by a loss of appetite and/or decreased food intake (anorexia of aging) that contributes to malnutrition, which in turn is associated with sarcopenia across the continuum of care [49, 71–76]. There is a high prevalence of the risk of malnutrition among older adults in various settings (i.e., community [27%], outpatient settings [31%], hospitals [46%], nursing homes and long-term care facilities [~ 50%]) [77]. In Singapore, approximately 30% of older adults are reported to be at nutritional risk in community and inpatient settings [78–80]. Importantly, the Singapore Longitudinal Aging Study reported that in Chinese older adults (65–90 years), malnutrition and risk of malnutrition were independently associated with sarcopenia after adjustment for age and gender [73].

When identifying malnutrition, we recommend the Global Leadership Initiative on Malnutrition’s two-step model, which advocates risk screening using any validated nutrition screening tool (e.g., MNA-SF [81], NRS-2002 [82] and MUST [83]), followed by diagnosis using at least one phenotypic (non-volitional weight loss, low body mass index or reduced muscle mass) and one etiological criterion (reduced food intake or assimilation, or inflammation, or disease burden) [84].

### Diagnosis of sarcopenia across the continuum of care

#### Recommendation 5

**In community and outpatient settings, bioimpedance analysis can be used to determine low muscle mass. In the absence of other alternatives, calf circumference can be used as a surrogate measure of muscle mass in patients without edema and not suspected to have sarcopenic obesity. In inpatient settings, dual-energy X-ray absorptiometry can be used as the reference standard for measuring muscle mass.**

We propose using bioimpedance analysis (BIA) to estimate appendicular skeletal muscle mass (ASM) in community and outpatient settings [8, 9] due to its affordability, portability and ease of use [8, 9, 48, 85]. In the absence of BIA, calf circumference can be used as a surrogate measure of muscle mass [9, 25] as it is associated with poor physical strength and is predictive of physical performance and survival in older adults [86, 87]. Caution is required in patients suspected to have edema or sarcopenic obesity as the use of calf circumference in these settings will give a false-negative result. Recommended cut-off values for BIA and calf circumference to identify low muscle mass based on the AWGS 2019 are presented in Table 3.

For inpatients, several consensuses recommend dual-energy X-ray absorptiometry (DXA) to quantify ASM [8, 9, 12, 25] as it serves as a ‘reference’ standard due to a balance of accuracy and accessibility in clinical practice [88]. We suggest using the AWGS-recommended cut-off values for the diagnosis of low ASM using DXA in confirming the diagnosis of sarcopenia (Table 3).

#### Recommendation 6

**Physical performance can be measured using the usual gait speed or by using the 5-times chair stand test with a cut-off of ≥12 s as a surrogate measure of gait speed of 1.0 m/s.**

Physical performance has recently been defined as an objectively measured whole body function related with mobility [60] and can be assessed by a variety of methods. The usual gait speed is a pragmatic and reliable measure of physical performance [8, 9, 25]. Gait speed is predictive of adverse clinical outcomes, such as falls, functional decline, cognitive decline and mortality in Asian populations [89–92]. We suggest using the AWGS 2019-recommended cut-off value for measuring low gait speed in the 6-m walk test (Table 3). Low gait speed is an indicator of severe sarcopenia when combined with the presence of low muscle strength and low muscle mass [9].

When it is not feasible to measure the usual gait speed, we recommend the 5CST as a surrogate measure of gait speed in community-dwelling older adults [93]. Based on the study by Nishimura et al., a gait speed of 1.0 m/s correlates with a cut-off of ≥12 s for 5CST [93]; this is also recommended by AWGS 2019 [26].

### Interventions for muscle health

#### Recommendation 7

**Progressive resistance/weight-based training is effective for improving muscle mass, strength and physical performance.**

In general, for older adults age 65 and above, more physical activity (frequency, duration and/or volume) leads to greater benefits as per the 2020 WHO Guidelines [94]. The International Clinical Practice Guidelines for Sarcopenia prescribes progressive RET as the first-line therapy for sarcopenia [25]. Similarly, the American College of Sport Medicine strongly recommends RET to increase strength and power in older adults [95]. Evidence has demonstrated the benefit of exercise training, especially RET for ≥3 months, in improving muscle mass, strength and gait speed in older adults [12, 70, 96–98]. Additionally, higher RET volume (total repetitions [number] x external load [kg]) is associated with greater improvements in LBM, with every additional 10 sets of exercise performed per session leading to an expected gain of 0.5 kg in LBM [97]. With each incremental increase in exercise intensity from low (< 60% 1-repetition maximum [RM]) to low/moderate (60-69% 1-RM), low/moderate (60-69% 1 RM) to moderate/high (70-79% 1-RM), and moderate/high (70-79% 1-RM) to high (> 80% 1 RM), a 5.5% increase in strength is expected [96, 99, 100].

Table 4 outlines an example of how a 12-week structured RET-based exercise program for older adults can be implemented in terms of specificity, overload and progression exercise prescription principles [95, 99, 101–104]. The application of moderate or greater intensity muscle strengthening exercises 2 or more days a week receives a strong recommendation with moderate certainty of evidence by the WHO Guidelines 2020 [94]. The Borg scale (Table 5) allows individuals to rate their level of exertion during exercise and can be used as a simplified alternative to gauge exercise intensity [104, 105].

**Table 4:** Example of a 12-week structured RET-based exercise program^a^

Week	1–2	3–4	5–6	7–8	9–10	11–12
Aim	Attain Adaptability		Develop Muscle Bulk		Build Strength	
Type [101]	Postural stabilization, Body weight Closed chain exercises			Free weights, open chain exercises		
Frequency (alternate days per week)	1–2	1–2	2	2	2–3	2–3
^b^Intensity (number of repetitions to fatigue) [101, 102]	20 low	15 low	12 moderate	10 moderate	8–10 moderate-high	6–8 high
Volume (number of sets)	1	1–2	2	2–3	2–3	3
Specific muscle groups	Core: *Abdominals* *Back* *Chest* Proximal Stabilisers: *Shoulders* *Hips* • Wall push ups • Bench presses • Crunches • Lunges • Mini Squats • Bridging		Distal Peripherals: *Arms* *Legs* Proximal Stabilisers: *Shoulders* *Hips* Core: *Abdominals* *Back* *Chest* • Resistance Bands • Weight Machine Stations • Arm Ergometry • Leg Pedal		Distal Peripheral: Arms Legs Proximal Stabilisers: *Shoulders* *Hips* Core: *Abdominals* *Back* *Chest* • Dumbbells • Weight Machine Stations	

^a^Adapted and modified from Peterson MD, Gordon PM. Resistance exercise for the aging adult: clinical implications and prescription guidelines. Am J Med 2011;124(3):194–8

^b^Progression of intensity – initially, the intensity of the exercise (weight or resistance loading) may be increased when the subject can achieve ≥20 repetitions in good form – indicative that current resistance is below the 60% 1RM threshold required for muscle strengthening [102, 103]. Progressively, the load can be adjusted higher to reflect higher intensity through the number of repetitions to fatigue or reduced ability to retain good form. Higher intensity RET can attain fatigue and should be stopped before strain of the training muscle

**Table 5:** The RPE Borg Scale

Borg’s Rating of Perceived Exertion (RPE) Scale^a^	
Perceived Exertion Rating	Description of Exertion
6	No exertion; sitting and resting
7	Extremely light
8	
9	Very light
10	
11	Light
12	
13	Somewhat hard
14	
15	Hard
16	
17	Very hard
18	
19	Extremely hard
20	Maximal exertion

^a^The scale is a guide as to how much older persons can push themselves during the resistance-based exercises. Exercises can be stopped or modified when the participants report perceived exertion levels beyond 15 or “Hard” on the Borg scale [105]

It is important to consider task-specific functional training in enhancing muscle health and function in older adults [102]. The principles of specificity, overload and progression should be applied similarly in the design. One example of such a program is the Lifestyle approach to reducing Falls through Exercise (LiFE), where strength training (with balance training) is embedded in daily activities [106]. This emphasis on functional balance and strength training is given strong recommendations with moderate certainty of evidence by the WHO Guidelines 2020 [94].

#### Recommendation 8

**Adequate calorie and protein diet support muscle health for healthy community-dwelling older adults.**

A total daily protein intake of at least 1.0–1.2 g/kg body weight is recommended in healthy individuals > 65 years of age [107]. In active, exercising healthy older adults, protein intake of ≥1.2 g/kg body weight/day is advised [107]. In addition, even distribution of protein intake throughout the day may help ensure some degree of muscle protein synthesis (MPS) throughout the day [107–112]. However, to maximize MPS, crossing an anabolic threshold may be necessary. Some studies suggest a threshold of 25–30 g protein per meal [107], whereas others suggest 0.4 g/kg/meal of protein [108]. Since the ideal consumption pattern remains to be elucidated [107], clinicians should evaluate and adjust the nutritional interventions from time to time. An adequate intake of protein needs to be accompanied by appropriate energy intake of 30 kcal/kg body weight/day as recommended for older adults. This value should be individualized based on clinical and patient factors [27]. In Singapore, HCPs can utilize the online calculator from the Health Promotion Board to determine the energy values and nutrition composition of various foods [113].

The WHO recommends oral supplemental nutrition (from food fortification to ONS) plus dietary advice for older adults with undernutrition in the community setting [20]. In older adults with chronic conditions with or at risk of malnutrition, ONS are strongly recommended when dietary counselling and food fortification is insufficient to meet nutritional goals [27]. Limited evidence also suggests that high protein ONS with HMB (HP-ONS + HMB; 1.5 g/day) may increase muscle strength in the presence of resistance exercise in community-dwelling older women [114].

#### Recommendation 9

**Supplementation of protein and calories, either via whole foods and/or high protein oral nutrition supplements, should be the primary focus of any nutrition interventions aimed at optimizing muscle health and recovery in hospitalized patients.**

Older adults with acute or chronic disease require a dietary protein intake of 1.2–1.5 g/kg body weight/day [107]. In severe illness, injury or severe malnutrition, up to 2.0 g protein/kg body weight/day may be necessary [107]. The total daily protein requirement can be divided evenly across three main meals to enable some degree of MPS throughout the day [107–112]. However, at least 25–30 g [107] or 0.4 g protein/kg [108] would be required to maximize MPS to account for increased anabolic resistance, periods of energy deficit and loss due to first-pass effect during digestion [107, 111, 115]. Older people with severe kidney disease (estimated glomerular filtration rate < 30 mL/min/1.73m^2^) and not on dialysis may need to limit protein intake [107]. In this scenario, the challenge lies in balancing the avoidance of excess protein intake to optimize renal health versus insufficient protein intake leading to protein energy wasting [116].

ONS may be offered to hospitalized patients to lower the risk of functional decline [27]. Upon meeting basic protein and caloric requirements with food, or when food intake alone is insufficient to meet increased nutritional requirements as specified above, additional supplementation may be considered using HP-ONS + HMB, HMB with arginine and glutamine (HMB-Arg-Glut) alone and/or leucine alone to improve muscle health. In older patients who are malnourished or at risk of malnutrition, limited evidence suggests that HP-ONS + HMB increases muscle strength [117] and LBM [118]. In contrast, a meta-analysis demonstrated that leucine supplementation significantly increases LBM and not muscle strength in patients with sarcopenia [119].

Some evidence suggests that the use of HMB 2–3 g per day may help prevent muscle mass loss in older adults on prolonged bed rest [120, 121]. Larger studies are required to confirm these findings.

#### Recommendation 10

**Meeting the recommended daily intake of vitamin D (600–800 IU) may improve muscle strength across the continuum of care and vitamin D deficiency should be treated.**

Vitamin D is vital for maintaining normal muscle function. In patients with sarcopenia, a target serum vitamin D of > 30 μg/L is recommended to optimize outcomes. A recent study in Singapore, which recruited community-dwelling older adults who are not at risk of malnutrition, reported the prevalence of vitamin D insufficiency (20–30 μg/L) and deficiency (< 20 μg/L) as 38.5 and 13.5%, respectively [122].

At present, routine serum vitamin D testing in the general population is not recommended. However, vitamin D supplementation has a small beneficial effect on muscle strength [123] and a daily intake of 600–800 IU of vitamin D is recommended for older adults [124]. In patients > 65 years who are vitamin D deficient, replacement with oral cholecalciferol 50,000 units weekly may be beneficial until the serum level is above 30 μg/L [125], particularly in the context of sarcopenia [126, 127].

#### Recommendation 11

**A combination of physical activity and nutritional interventions is strongly recommended for optimal muscle health in patients with malnutrition or at risk of malnutrition.**

Combining exercise and nutrition is an effective therapeutic intervention for sarcopenia [25] and for improving muscle health in older adults with or at risk of malnutrition [27]. Protein supplementation augments muscle mass and strength gains from prolonged RET in older adults [128]. A meta-analysis found that protein supplementation has a stronger effect in preventing loss of muscle mass and leg strength in older adults at risk of sarcopenia and frailty compared with RET alone [129]. There is also some evidence of the additive benefit of RET and vitamin D3 supplementation for muscle strength in older individuals [130]. In addition, a recent small, four-arm, randomized controlled trial in Japan demonstrated the synergistic effect of RET, protein and vitamin D supplementation in older adults with sarcopenia or dynapenia, with significant improvements in lower limb muscle quality and strength, when compared with exercise alone, nutritional supplementation alone or the control group [131]. A significant improvement in terms of appendicular lean muscle mass was also seen in subjects who started the trial with low muscle mass in the combined intervention group [131].

Performing exercise in close temporal proximity to the nutrition intervention or protein-rich meal has been shown to be beneficial for muscle anabolism [132]. Physical activity improves the sensitivity of MPS response to the provision of amino acids and reduces anabolic resistance. This enhanced response is sustained for days after resistance-based training [133].

### Overall management

#### Recommendation 12

**A multidisciplinary team (physicians, dietitians/nutritionists, physiotherapists, nurses and other relevant healthcare professionals) is essential for optimizing muscle health in all settings.**

A multidisciplinary team (MDT) facilitates individualized and patient-centric management of muscle health [25] and has been shown to increase the frequency of evaluation of sarcopenia and implementation of rehabilitation nutrition in Japan [134]. In Singapore, geriatricians and rehabilitation medicine physicians routinely lead MDTs in hospital settings. Family physicians would be well poised to do the same in the community setting. Lifestyle, biological and psychosocial factors that influence muscle health should also be identified and managed by various HCPs [7], further iterating the importance of the MDT.

#### Recommendation 13

**Patients and caregivers should be educated on the importance of combining physical activity and nutritional interventions for improving muscle health.**

Patient and caregiver education should emphasize that the treatment for sarcopenia requires RET and optimal nutritional intake [25]. Improving patient awareness on the causes of and strategies to reverse or mitigate muscle impairment may address any misconceptions about sarcopenia as an inevitable consequence of aging and improve adherence to prescribed interventions [25]. In addition, acceptability of RET may be enhanced by knowledge that using low weights at higher velocity can also be beneficial [135] and is supported by the WHO 2020 Guidelines [94].

We suggest several population-based strategies to further encourage muscle health in Singapore (Table 6) [136].

**Table 6:** Suggested population-based strategies to promote muscle health in Singapore [136]

Population-based strategies	Examples relevant to promoting muscle health
Screening and outreach	• Identify early muscle loss by detecting deterioration in usual physical function or strength, during screening events or visits to polyclinics or general practice clinics. • Improve HCP awareness of muscle loss identification by mere observation of general body muscle bulk in older individuals over time.
Referral and follow-up	• Encourage older persons to focus on resistance exercises in order to build muscle strength and mass, e.g., in fitness corners, resistance exercise parks and senior-friendly gyms. • Active referral of older persons to available community resources, e.g., day rehabilitation centres, as appropriate. • Consider referral to a dietician, if the older adult is malnourished and might require more complex interventions.
Health teaching and coaching	• Emphasize the importance of optimal calories (30 kcal/kg body weight/day) and protein (≥1.2 g/kg body weight/day) in older adults. • Promote more active uptake of resistance exercise by advocating for gradual progression with sufficient intensity.
Consultation and collaboration	• Collaborate with organizations to include basic nutritional literacy and simple resistance exercise training as part of the nursing skills requirement for foreign domestic workers, particularly those tasked with caring for older adults.
Advocacy and policy development	• Make government subsidies available via the Senior Mobility Fund to support rehabilitation programs at home (e.g., telerehabilitation equipment, weights and resistance bands). • Currently, eligible patients can already access subsidy for ONS under the Senior Mobility Fund.
Social marketing	• Link exercise and nutrition for muscle health with campaigns related to bone health – i.e., ‘to prevent fractures and falls, take calcium for bone and protein for muscle.’

#### Recommendation 14

**Across all healthcare settings, urgent initiatives are required to raise awareness, educate and skill relevant healthcare professionals on screening, prevention and management of poor muscle health.**

Considering the known gaps and lack of standardization in clinical practice [20, 23], HCPs across all healthcare settings need to be trained in the assessment of muscle strength, muscle mass and physical performance, and in the prevention and management of poor muscle health across all settings [51]. In this context, these recommendations can guide best practices among HCPs.

## Discussion

To optimize muscle health in older adults in Singapore, we have proposed evidence-based practical guidance on screening, diagnosis and multi-modal interventions encompassing RET and nutrition across the continuum of care. Our concise and harmonized recommendations highlight the importance of a holistic multidisciplinary approach to muscle health in the older adults, and provides a unique, explicit, evidence-based progressive RET protocol as an intervention to improve muscle strength and mass. While tailored for the Singapore population, these recommendations may also be applicable in similar countries across Asia.

In the AWGS 2019 recommendations, we have noted that handgrip strength is the sole measure of strength [26]. For some older patients, they may not be able to operate the hand dynamometer to the required standard due to physical and/or cognitive limitations. Our inclusion of the 5CST ≥10s as a surrogate measure of lower limb strength based on Asian data from Makizako et al. [54] will facilitate the measurement of strength in this group of patients. We believe that this small but important difference will contribute to a more streamlined and comprehensive assessment of Asian patients at risk of poor muscle health.

In addition, our consensus statement on physical interventions can be utilized as part of a cost-effective targeted communication campaign promoting physical activity in older adults, and would be in keeping with the recommendations of the WHO Guidelines 2020 [94] as well as the WHO Global Action Plan on Physical Activity 2018–2030 [137].

In order to implement these recommendations in clinical practice, comprehensive education of HCPs on the importance of muscle health and potential deleterious consequences of sarcopenia is required. Urgent initiatives are hence needed to train relevant HCPs to screen and diagnose sarcopenia; utilize evidence-based exercise and nutrition interventions for muscle health; and monitor the outcomes of these interventions.

We acknowledge that our recommendations are based on a narrative review and should be used in this context. However, we have included the latest available literature, international guidelines and results of systematic reviews on muscle health to guide our recommendations.

## Conclusion

The rapidly aging population in Singapore and Asia necessitates the prioritization of muscle health from a public health perspective, and strong collaboration among various stakeholders, including clinicians, professional societies and policy makers are urgently required. Therefore, we hope that these consensus recommendations on muscle health can serve as a holistic platform for the comprehensive advancement of muscle health, and spur much needed new research in this area to fill the gaps of knowledge that still remains.

## Data Availability

Not applicable.

## Abbreviations

5CST: 5-times chair stand test
ASM: Appendicular skeletal muscle mass
ASMI: Appendicular skeletal muscle index
AWGS: Asian Working Group for Sarcopenia
BIA: Bioimpedance analysis
DXA: Dual-energy X-ray absorptiometry
EWGSOP: European Working Group on Sarcopenia in Older People
HCP: Healthcare professional
HMB: β-hydroxy β-methylbutyrate
HP-ONS: High protein oral nutritional supplements
LBM: Lean body mass
MDT: Multidisciplinary team
MPS: Muscle protein synthesis
ONS: Oral nutritional supplements
RET: Resistance exercise training
RM: Repetition maximum
WHO: World Health Organization
